# Cryopreservation of Ovarian Tissue: Opportunities Beyond Fertility Preservation and a Positive View Into the Future

**DOI:** 10.3389/fendo.2018.00347

**Published:** 2018-06-28

**Authors:** Stine G. Kristensen, Claus Y. Andersen

**Affiliations:** Laboratory of Reproductive Biology, Faculty of Health Science, The Juliane Marie Centre for Women, Children and Reproduction, University Hospital of Copenhagen, University of Copenhagen, Copenhagen, Denmark

**Keywords:** ovarian tissue cryopreservation, fertility preservation, cell/tissue-based hormone replacement therapy, induction of puberty, ovarian resection, social freezing, *in vitro* maturation

## Abstract

In current years, ovarian tissue cryopreservation (OTC) and transplantation is gaining ground as a successful method of preserving fertility in young women with primarily cancer diseases, hereby giving them a chance of becoming biological mothers later on. However, OTC preserves more than just the reproductive potential; it restores the ovarian endocrine function and thus the entire female reproductive cycle with natural levels of essential hormones. In a female population with an increased prevalence in the loss of ovarian function due to induced primary ovarian insufficiency (POI) and aging, there is now, a need to develop new treatments and provide new opportunities to utilize the enormous surplus of follicles that most females are born with and overcome major health issues associated with the lack of ovarian hormones. Cell/tissue-based hormone replacement therapy (cHRT) by the use of stored ovarian tissue could be one such option comprising both induction of puberty in prepubertal POI girls, treatment of POI and premature menopause, and as primary prevention at the onset of menopause. In the current review, we explore known and entirely new applications for the potential utilization of OTC including cHRT, social freezing, culture of immature oocytes, and a modern ovarian resection for women with polycystic ovaries, and discuss the indications hereof.

## Introduction

The follicle constitutes the functional unit of the ovary and produces steroids and peptide hormones to regulate the female reproductive cycle. During the follicular phase more than 90% of the available oestradiol is produced by a single selected preovulatory follicle, which in the luteal phase is transformed to the corpus luteum that secretes progesterone in high concentrations. The unique physical distribution of the follicular reserve within the ovary, with the vast majority of small resting follicles located in the outer cortical region and the growing stages of follicles located in the inner medullary region, represents a perfect opportunity to preserve an organ function without freezing the entire organ. By isolating the cortical region, containing 90% of the follicular reserve, human ovarian tissue has been successfully cryopreserved for fertility preservation in young women with cancer diseases for over two decades. Subsequent transplantation of thawed ovarian tissue has restored ovarian endocrine function in 95% of the patients and resulted in the birth of over 130 children worldwide (1, 2). Half of all the children born have been conceived by natural conception, which highlights the exceptional ability of this procedure to actually restore an entire organ function in contrast to conventional fertility preserving strategies like oocyte and embryo freezing, in which the fertility potential is fixed by the number of oocytes retrieved and IVF is the only option to conceive. Moreover, ovarian tissue cryopreservation (OTC) can be performed from day to day and is therefore the only fertility preserving strategy for girls and young women who needs to undertake acute gonadotoxic treatment, and for prepubertal girls who do not yet produce mature oocytes for freezing.

Primary ovarian insufficiency (POI) is defined as the cessation of the ovarian function before the age of 40 years, and is a common cause of infertility in women. Its incidence is estimated to be as high as 1 in 100 by the age of 40, and 1 in 1,000 by the age of 30 years (3, 4). Spontaneous POI include genetic, autoimmunological, inflammatory, enzyme deficiency, metabolic, or very often idiopathic causes, whereas induced POI occurs mainly due to oncological treatment such as surgery (bilateral oophorectomy), chemotherapy and radiotherapy (5). POI not only interferes with a woman's reproductive potential, but the cessation of sex steroid production is also associated with an increased risk of osteoporosis, cardiovascular disease, earlier mortality, obesity, sexual dysfunction, dementia, and cognitive decline (6–8).

**Figure 1:**
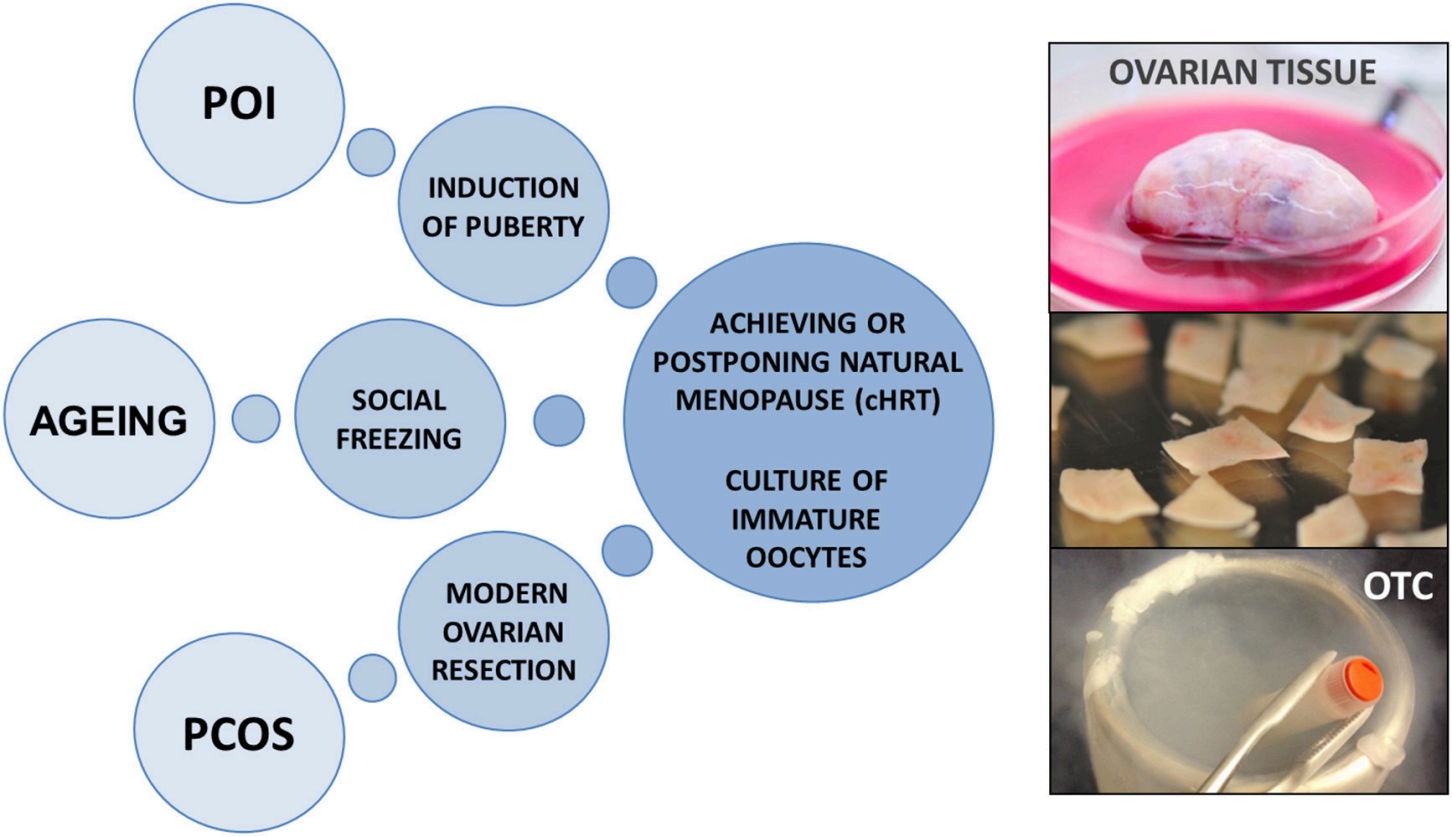
Potential applications for ovarian tissue cryopreservation beyond fertility preservation in young women with cancer diseases. OTC, ovarian tissue cryopreservation; cHRT, cell/tissue-based hormone replacement therapy; POI, premature ovarian insufficiency; PCOS, polycystic ovary syndrome.

Up until now fertility preservation has been the primary goal of OTC in young girls and women diagnosed with a malignancy or genetic disease threatening to destroy their ovarian reserve. However, the restoration of an organ function, the steroidogenic capacity of the transplanted ovarian tissue and the unique access to immature oocytes from small antral follicles have now evoked new perspectives and ideas to expand the utilization of OTC beyond its traditional purpose of fertility preservation for medical reasons. These novel ideas approach a broader population of women and include utilization of OTC for cell/tissue-based hormone replacement therapy (cHRT), non-medical reasons, optimizing culture systems for immature oocytes, and performing a modern ovarian resection for women with polycystic ovaries (Figure 1). This kind of ideas which are technical possible, but not yet proven, will undoubtedly raise controversy in the field and a plethora of questions concerning the ethics, safety, cost-effectiveness, superiority, and implications of the proposed procedures. In this paper, we aim to explore known and entirely new applications for the utilization of OTC and discuss the indications for such procedures.

## Cell/tissue-based hormone replacement therapy (cHRT)

More efficient cancer treatments combined with a general increase in life expectancy have led to a higher frequency of POI in a growing population of cancer survivors and aging women. It is widely accepted that the pillar of treatment of POI is pharmacologic hormone replacement therapy (pHRT), at least until the average age of natural menopause. Pharmacologic HRT with estrogen alone or estrogen and progestogens in combination is known to effectively compensate for the loss of ovarian hormone production, but only when it is delivered at an optimal dosage, frequency, and at an appropriate time (8, 9). Unfortunately, pharmacological delivery methods of HRT are not integrated into the hypothalamic-pituitary (HP) axis which would facilitate feedback and regulation of dosage and timing of circulating hormone levels. As such, pharmacological hormone delivery results in consistently higher serum concentrations as compared to those associated with normal functional ovaries.

New approaches using cHRT offer a potential physiological solution to timely control of hormone delivery and the ability to restore the functionality of the HPO axis. OTC can be used as cHRT as thawed ovarian tissue grafted into the pelvis cavity restores the natural hormone milieu and endocrine function of the ovary which have been consistently documented by a rise in estradiol and a decrease in FSH and LH levels returning to premenopausal levels 4–5 months after transplantation leading to cessation of menopausal symptoms and renewed menstrual cycles in the vast majority of transplanted patients (1, 10, 11). Restored hormone production may even be the desired effect rather than fertility restoration in some patients (10), and a more appealing alternative to pHRT for some women. In Denmark around a dozen young women who entered menopause due to cancer treatment have had frozen/thawed tissue transplanted only to become a “normal woman” again and avoid menopause (10), The average duration of graft function is approximately 5 years, but the function can persist for over 9 years (10), depending on the follicular density at the time of OTC (2). The transplantation procedure may be repeated multiple times in order to stretch the longevity of the tissue, if sufficient amounts of ovarian tissue has been stored, hereby extending the lifespan of ovarian endocrine activity up to 11 years or more (12, 13). If patients are merely in the need of sex steroid production, a heterotopic graft site like the abdominal wall or a subcutaneous site may be preferred over an orthotopic site as it requires less invasive surgery and would obviate the need for general anesthesia and a laparoscopic procedure. A cHRT approach combining a heterotopic graft site with multiple transplantations extending the longevity of banked ovarian tissue can therefore facilitate prolonged ovarian endocrine function and have implications way beyond fertility preservation.

### Inducing puberty in pre-pubertal POI girls

Survivors of childhood cancer represent a rapidly growing population of patients in which temporary or permanent POI is a common side effect of the cytotoxic treatments. In addition, POI often has a genetic background with more than 50 genes in which mutations can be causative and many other genes that may be implicated (14). These genes can affect various processes such as gonadal development, DNA replication/ meiosis and DNA repair, hormonal signaling, immune function, and metabolism, and include conditions like Turner syndrome, sickle cell anemia, thalassemia, and galactosemia. These young girls often experience delayed puberty or need pHRT to induce puberty to enable the pubertal growth spurt as well as development of secondary sexual characteristics. However, pharmacological delivery of increasing doses of estrogen followed with progesterone only address some aspects of puberty and does not completely match the physiological complexity of the hormonal milieu during puberty. Moreover, there is only limited data on the long-term safety of exogenous hormones in childhood cancer survivors and pHRT can cause potentially significant side effects (15, 16).

Cell/tissue-based HRT can therefore be an attractive approach to provide a more physiological hormonal milieu for induction of puberty in pre-pubertal POI girls. In 2012, Poirot and co-workers published the first case report showing that stored ovarian tissue from pre-pubertal girls can provide adequate endogenous sex steroid hormone levels to induce puberty (17). In this case, a 13-year old girl developed POI after being treated for sickle-cell disease at the age of 10, and after having 3 out of 23 stored ovarian pieces transplanted, she entered puberty. A second case report was published shortly after with another 13-year old girl who had developed POI following treatment for Ewing sarcoma at the age of 9 (18). In both cases, a unilateral oophorectomy was performed before treatment and a small proportion of the frozen ovarian cortical fragments (i.e., 10–20% of the stored tissue) were transplanted to a heterotopic location in the former case and to the remaining ovary in the latter. Puberty was successfully induced in both girls although the graft function failed after nearly 2 years.

It can be discussed whether or not young girls should choose to save their ovarian tissue for later use to achieve fertility instead (19), but using only a small percentage (i.e., 10–20%) of the stored tissue might be worth it for some young girls to feel “normal” during a critical period of their emotional and social life as the physical maturation process directly affects body and brain to alter children's needs, interests, and moods. Several studies have suggested that young childhood cancer survivors with POI face significant psychosexual dysfunction and that the symptoms of POI could severely stunt social growth and exacerbate anxiety and feelings of isolation (20, 21). Therefore, cHRT could potentially be beneficial in the long run at both the physiological and psychological level, and the preferred choice for some pre-pubertal POI girls in order for them to experience the life of every other teenager and becoming a woman with physiological levels of sex hormones. However, the preferred treatment for young girls with POI is still pHRT, as it is cheap, simple, and ready available and does not include any surgical intervention. Moreover, the theoretical superiority of physiological hormone levels over the pharmacological levels for induction of puberty has not been proven in any human clinical trials and awaits further research.

### Achieving or postponing natural menopause

One century ago the average life expectancy corresponded to the natural age of menopause around the age of 50 years, however, nowadays the majority of women live beyond 80 years in many developed countries, and the demographic structure is changing toward an increasingly aging population in which women will spend 30–40% of their lives being postmenopausal. In combination with the increasing population of cancer survivors experiencing POI these developments in society calls for preventive strategies to alleviate and decrease short- and long-term consequences and health risks associated with the lack of ovarian endocrine function.

The use of pHRT has been vigorously debated for several decades. In the 1980's and 90's pHRT was administered to millions of women to relieve menopausal symptoms and by the mid-1990s estrogen became the biggest-selling prescription drug in the US. However, it all changed with the publication of the results from the Women's Health Initiative (WHI) randomized clinical trials in 2002 (22), suggesting pHRT to cause an increased risk of breast cancer, after which many women discontinued pHRT or avoided starting pHRT at all ages, including before age 50 years (23, 24). One of the big misunderstandings was that the results from the WHI studies were inappropriately extrapolated from women in the late postmenopausal stage (aged >60 years) to younger women in the early stage of postmenopause (50–59 years), and even further to women experiencing premature or early natural menopause. The WHI study was built upon numerous observational studies and clinical trials consistently demonstrating benefits in the prevention of chronic diseases, which include reduced coronary heart disease (CHD) and mortality, when pHRT was initiated near the onset of menopause (25–30). The WHI study was therefore designed to test the effects of pHRT in women initiating treatment a decade or more after menopause. This is where the “timing hypothesis” comes into play and suggest that different clinical effects occurs if hormones are initiated close to the onset of menopause compared with several years later (31). Basic and animal studies together with clinical studies have now shown that the timing of pHRT can be crucial with respect to especially CHD, cognitive decline, and dementia (8, 31–34). In 2005, the Multi-Institutional Research on Alzheimer Genetic Epidemiology study showed that the risk of dementia was reduced in women who initiated hormonal therapy at age 50–63 years, but was not reduced in women who started hormonal treatment at ages 64–71 or 72–99 years (35).

Reassessment of clinical trials in women initiating pHRT close to the onset of menopause together with more recent studies and meta-analyses now show a long list of benefits with estrogen alone therapy, and risks are considered rare. Beyond symptomatic relief, improvements in quality of life and a reduction in osteoporosis, estrogen-based therapy has now been shown to be cost-effective and have a very favorable risk–benefit profile in healthy women under the age of 60. Specifically, estrogen alone therapy have been shown to consistently decrease CHD in women under 60 years of age by up to 40% and to decrease all-cause mortality by 20–40% (36–38). Moreover, a long-term follow up on the WHI study showed that estrogen alone therapy resulted in a significant decrease in the total risk of cancer by 20% in women aged 50–59 years (39).

The majority of studies show benefits with estrogen alone rather than with estrogen plus progestogen, however, no particular HRT regimen can currently be advocated (31). Moreover, the use of age-appropriate estrogen doses has been reported to be crucial to maximize cardiovascular benefits while minimizing risk of adverse effects such as venous thromboembolism and stroke (32). This is where cHRT comes into the picture and we hypothesize that transplantation of stored ovarian tissue could be used to restore endocrine ovarian function at the onset of menopause or for women entering menopause prematurely. In 2015, we proposed that ovarian tissue frozen in the young years could be transplanted back at the time of menopause to provide continued endogenous, physiological levels of steroid production to postpone menopause (40). A small portion of stored ovarian tissue may be transplanted subcutaneously during local anesthesia multiple times providing menstrual cycles for a prolonged period, using the woman's own tissue and follicles to sustain menstrual cycles with natural variations in the whole armamentarium of hormones. In this way, natural fertility will also be avoided and prevent senior women to conceive spontaneously. Recent animal studies have demonstrated that supraphysiological plasma levels of estrogen were required for pHRT to achieve benefits in bone health that were comparable to those achieved by cHRT (using ovarian cell constructs) at much lower plasma hormone concentrations (41). Such studies suggest a potential benefit of physiological secreted sex steroid hormones under the control of the HPO axis at which effects can be obtained at lower and safer plasma levels.

In theory, cHRT using OTC at a young age could be used as a physiological solution to prevent the massive medical legacy of osteoporosis and menopause-related conditions in the large population of aging women, however, currently the majority of women would probably not subject themselves to an elective surgery purely for delaying menopause and it can be argued whether or not any women would prefer to have regular menstrual cycles up to 60 years of age, when readily available pHRT compounds can deliver the hormones without the bleeding. Nevertheless, OTC could potentially be justified in some women already undergoing pelvic surgery for other reasons for example, a Caesarean section or an appendectomy where ovarian tissue could be collected and frozen as an adjunct. This is, however, also an approach which is highly debatable and may not be ethically and medically appropriate as no studies have been conducted that directly or indirectly compare the benefit and disadvantage of ovarian tissue removal for future cHRT.

The group of women for whom the use of cHRT are most likely to become a clinical option initially is the thousands of women worldwide who have already had ovarian tissue frozen for fertility preservation due to different malignancies and genetic conditions. For various reasons, a large number of these women will not have used their stored tissue by the time they reach menopause, either prematurely or at the natural age of menopause. Some of these women might wish to use their store tissue for postponing menopause or to achieve natural menopause which is nowadays recommended by many scientific societies (8, 31). Studies have shown that women who become postmenopausal before the age of 50 years and do not receive any treatment have an increased risk for cardiovascular disease-related mortality compared to women receiving pHRT (42–46). Other studies have shown an almost doubled long-term risk of cognitive impairment or dementia in women who underwent oophorectomy before menopause (47). A risk that was eliminated if estrogen therapy was initiated after the surgery and continued up to age 50 years or longer (47), which is advertising a need for sustained ovarian function up to at least the age of natural menopause.

However, many concerns and unanswered questions exist; Is it safe? To whom is the risk-benefit profile actually favorable? Are physiological hormones better than pharmaceutical? Careful consideration of risks and benefits, individually structured counseling and close monitoring are needed for each woman who may want to use their ovarian tissue for primary prevention or postponing menopause. To summarize, cHRT in the form of stored ovarian tissue or ovarian cell constructs (41, 48, 49) could potentially be used at the onset of natural or induced menopause as an ideal time to institute preventative strategies that could potentially increase the quality and length of women's lives. However, this is again technical possible but not proven in a clinical setting with women in this age group, and it can be argued that pHRT should be preferred in all cases as it is the cheapest and simplest way to provide hormones, and no real evidence for the superiority of physiological hormone levels exists. Therefore, more research in the area of physiological and pharmacological hormones is needed in order for this potential application to be adopted clinically in the future.

## OTC for non-medical reasons

In many countries women also have the opportunity to preserve their fertility due to personal reasons and hereby postpone childbearing. The increasing age of childbearing observed in most high-income countries is often depicted as a result of women selectively choosing to pursue a career and other life goals before having children, however, evidence now suggests that the primary reason is the lack of a partner who is willing to commit to parenthood (50, 51). The traditional method of fertility preservation for these social indications is vitrification of mature oocytes. In recent studies, it has become evident that most of the women who cryopreserve oocytes for non-medical reasons are in their mid to late 30 s, well educated, socio-economically advantaged and single (52–54). Data concerning the reproductive experiences and outcomes of this growing group of women is still very limited, but two severe issues with today's policy for non-medical oocyte freezing have emerged. First of all, women are too old when they decide to store oocytes as the mean age at the time of freezing is around 37 years (53, 55), which may be deemed suboptimal (56). Additionally, one in five patients in this group is a low responder and combined with the advanced maternal age this results in one quarter of the women having fewer than 8–10 mature oocytes frozen which is considered necessary for a reasonable chance of success (57). The second issue is that the majority of the women with frozen oocytes do not come back to collect them and the utilization rate of the stored oocytes is currently below 10% (53, 55). Collectively, it appears that especially reproductive aged women may only to a limited extend benefit from this approach.

A proposition would be to use OTC as an alternative to oocyte vitrification in connection with fertility preservation for non-medical reasons. In contrast to mature oocytes, transplanted ovarian tissue will give the woman the opportunity to conceive spontaneously without IVF, which many women and their potential future partners would probably appreciate. If the woman ends up not needing the stored ovarian tissue for fertility, then the tissue is not wasted, but could instead be used as cHRT to alleviate postmenopausal symptoms later on or avoiding POI. Thus, OTC may serve multiple purposes depending on the need for either fertility or endocrine function and provide a better justification of such intervention. In addition, by storing ovarian tissue, and not just a fixed number of mature oocytes, a range of potential new treatments, which are currently being developed, might become available for these patients in the future. These treatments include *in vitro* follicle activation (58), culture of human preantral follicles (48, 59, 60), autologous transfer of mitochondria to oocytes (61), and *in vitro* maturation of immature oocytes (62).

OTC requires at least two surgical procedures to collect and graft ovarian tissue and it might be overwhelming and excessive for some patients. Therefore, OTC may only be a preferable option over oocyte vitrification for some groups of patients in particular women of advanced maternal age with a low ovarian reserve or poor responders which require multiple cycles of controlled ovarian stimulation. For these groups of patients, their future use of stored ovarian tissue could potentially be enhanced by *in vitro* follicle activation (IVA) in which the resting pool of follicles is activated mechanically and/or biochemically prior to transplantation. In 2013, Kawamura et al. were the first to test IVA in a clinical setting and enabled POI patients without menstrual cycles for several years to conceive with their own oocytes by activating a residual pool of resting follicles (58). This technique has so far resulted in three live births in Japan and two pregnancies in other clinics (58, 63–65).

Taken together, oocyte cryopreservation is the clinical available option for non-medical freezing, but it can be argued that women seeking fertility preservation for non-medical reasons should be presented with both fertility preserving options and the potential beneficial applications for cHRT and future use, and then the woman can help decide which method is most suitable for her. It should be highlighted that age is the most limiting and critical factor for both methods, and the success rate always depends hereof, which means that the issue with low ovarian reserve will apply for both methods, and low return rates are probably an inevitable factor in this group of women.

## A modern ovarian resection for anovulatory PCOS women

A completely new way of utilizing OTC is in the context of surgical treatment for anovulatory women with polycystic ovaries. Polycystic ovary syndrome (PCOS) is one of the most common endocrine disorders among women in their reproductive age and is a predominant cause of anovulatory infertility. In 1930s, Stein and Leventhal developed the ovarian wedge resection (OWR) in which the ovarian volume was surgically reduced during laparotomy (66). For decades OWR was the only treatment for PCOS as it effectively restored regular menstrual cyclicity for a period of time in 80–90% of women with PCOS and allowed conception in 45–65% of those who underwent surgery (66). However, the procedure later became less popular due to significant side effects; primarily a significant incidence of postoperative pelvic adhesion formation adding to the infertility-issue (67–69). Furthermore, follow-up studies revealed that OWR was not always curative and a significant number of women, 30–35%, reverted into a state of anovulation or oligo-ovulation (69, 70). With the introduction of clomiphene citrate (CC) and human menopausal gonadotropins in the 1960s the surgical treatment of PCOS was less used, and today CC is used as the first-line therapy for ovulation induction in women with anovulatory PCOS (71, 72). However, 10–15% of women are CC-resistant and will not ovulate in response to CC, and in those who do respond, not all will conceive. Laparoscopic ovarian drilling (LOD) using a unipolar electrode is currently recommended as a successful second-line treatment for ovulation induction in women with CC-resistant PCOS, as it has been shown to be just as effective as gonadotropin treatment, but less expensive and not associated with an increased risk of multiple pregnancy, ovarian hyperstimulation syndrome (OHSS) or pregnancy loss (71, 73–75). In the 1980's the first studies on LOD showed that ovulation was restored in 92% of patients, with a pregnancy rate of up to 80% (76). Furthermore LOD allows multiple attempts of conception, but without intensive monitoring, and is preferred by the majority of patients (75). The main shortcomings of LOD are postoperative adhesion formations and the potential risk of affecting the ovarian reserve in case of excessive damage (75).

We therefore hypothesize that the development of a modern version of an ovarian resection in which ovarian biopsies are excised surgically during laparoscopy and subsequently frozen could potentially provide an alternative to traditional LOD and OWR in CC-resistant women with PCOS. In this case, the surgical intervention should be performed in a way in which whole pieces of cortical and medullary tissue is removed in amounts similar to what is destroyed during LOD, and the ovarian cortex could then be isolated and frozen to preserve the follicles for potential later use instead of wasting them when performing LOD or OWR. Thus, in cases were too much tissue is removed during the surgical procedure the stored ovarian tissue can be grafted as back-up, or potentially be used for cHRT if the patient later on enters menopause prematurely or for fertility at an advanced reproductive age. In 1970's it was incidentally noted that ovarian biopsy alone (taken for diagnostic purposes) could induce regular menstrual cyclicity and conception in some women with PCOS (77), and in 1980's OWR was also performed through multiple ovarian biopsies and reported successful using a laparoscopic approach with less adhesion formations (78). Like LOD the proposed modern version of an ovarian resection through excision of ovarian biopsies would restore regular ovulation and allow spontaneous pregnancy, and in patients who remain anovulatory following the procedure most of them would have an increased responsiveness of the ovary to CC-treatment or respond less aggressively to exogenous hormone stimulation. Taken together, the proposed procedure using OTC to advance a surgical treatment for PCOS patients is completely theoretical and has not been tested in a clinical setting, however, this approach could potentially utilize the excessive—and in this case harmful—follicle pool in a beneficial way by relieving PCOS symptoms immediately and at the same time securing ovarian endocrine function later on in life. The efficacy and advantages of the proposed procedure now needs to be proven and compared to LOD in a clinical setting to justify any potential use in the future.

## Optimizing culture systems for immature oocytes

By freezing ovarian cortical tissue for fertility preservation, it is possible to gain access to a wide range of immature oocytes, including early preantral and small antral stage follicles, which can be isolated and collected from surplus or donated ovarian tissues (62, 79). This provides a rare opportunity to characterize the basic molecular mechanisms controlling and regulating human follicular growth and maturation in unstimulated ovaries of fertile women (80, 81), and to develop and optimize culture conditions for immature oocytes (59, 62), which could provide additional fertility for young women with a wide range of indications. Thus, utilization of immature oocytes could in this way augment the overall fertility from the OTC procedure. Moreover, young cancer survivors with a risk of ovarian involvement from the underlying cancer cannot have their stored ovarian tissue transplanted safely, and current advances in culture systems for the earliest stages of follicles could potentially provide fertility for these women in the future. In a recent paper, Telfer et al. succeeded with *in vitro* growth of unilaminar follicles (IVG) in a multi-step culture model which supported the development and maturation to the Metaphase II stage (60).

Several studies have now shown that a considerable number of immature oocytes can be collected from small antral follicles visible on the surface of the ovary or released to the dissection medium during the preparation for OTC (62, 82–84). These oocytes can be matured, vitrified and used to augment fertility to the patient (62, 84–86). The maturation rate has been shown to vary between ~30 and 60% (87), and the first three live births resulting from a cryopreserved embryo obtained from *in vitro* matured (IVM) oocytes has been reported (82, 88, 89). However, there is plenty of room for improvement and currently IVM is mainly used as an additional fertility preserving option in combination with OTC or to avoid OHSS in PCOS patient. A more widely use of IVM is currently not accepted due to the fact that implantation and developmental potential of embryos derived from IVM oocytes has consistently been reported to be significantly lower in comparison to *in vivo* matured oocytes (90, 91).

One explanation for the inefficiency of IVM is that IVM protocols have changed little, if any, since the first reported birth obtained by IVM oocytes in 1994 using a non-specific medium (MEM) with addition of FSH, LH and estradiol as hormones and maternal serum as supplements (92). In recent reports, commercial maturation media have been used for IVM in both patients with PCOS and women undergoing fertility preservation, but there is no real evidence that the formula is appropriate for oocyte maturation *in vitro* (91). Thus, IVM in connection with OTC provides a unique platform to compare multiple culture media compositions and improve basal maturation conditions for IVM. Another explanation for the decreased implantation and pregnancy rate with the use of IVM derived embryos is insufficient endometrial receptivity. The limited time between oocyte recovery and embryo transfer is potentially insufficient to allow full completion of the endometrial proliferative phase, which could compromise the formation of a secretory endometrium and the chances of embryonic implantation (91). A study by De Vos et al. has shown that implantation and pregnancy rates were much higher in replacement cycles using frozen embryos obtained after IVM compared to cycles in which embryos were transferred fresh after IVM (93). Thus, the relatively lower clinical efficiency that currently characterizes IVM might derive not exclusively from reduced oocyte quality caused by inadequate IVM conditions, but also from inadequate endometrial receptivity. Therefore, the notion that IVM oocytes are intrinsically less developmentally competent will need to be reconsidered.

Despite the current limitations, IVM continue to attract growing interest in consideration of potential novel applications in human ART. However, to advance more sophisticated IVM systems able to reproduce more physiologically and efficiently the process of oocyte maturation, we need more research material and settings such as provided by OTC, in which we can gain a better understanding of the biological mechanisms transforming a fully-grown oocyte into a mature fertilizable gamete.

## Conclusion

OTC in prepubertal girls and young women is becoming an increasingly well-established method of fertility preservation in many clinics worldwide. However, by storing ovarian tissue more than just the reproductive potential is preserved. The steroidogenic capacity and endocrine function of the tissue could potentially expand the utilization of OTC and the ovarian reserve to other beneficial applications throughout the female lifespan and several indications beyond cancer. With an increasing prevalence in loss of ovarian function due to POI and aging we now need to explore these opportunities and outweigh the cost-benefit and risk-benefit ratios associated with potential new treatments. Nonetheless, it is important to recognize that fertility and endocrine function restored with ovarian tissue go hand in hand and that both are the result of follicular activity.

In conclusion, the described and suggested applications for OTC in this review are technically possible, however they have not been validated clinically (with the exception of puberty induction), and therefore the suggested applications should be regarded as an optimistic view on the potential future use of OTC. Moreover, the suggested applications for OTC are thought provoking to many people and the controversial opinions hereof should of course be recognized. Finally, the burden of repeated surgical interventions in connection with OTC must in all cases be weighed against the simple, cheap, and readily available pHRT medications which already exist and are administered to millions of women worldwide.

## Author contributions

SK and CA both contributed to the conception and writing of this paper.

### Conflict of interest statement

The authors declare that the research was conducted in the absence of any commercial or financial relationships that could be construed as a potential conflict of interest.
